# Induction of Labor in Primigravid Term Pregnancy with Misoprostol or Dinoprostone: A Comparative Study

**DOI:** 10.7759/cureus.5739

**Published:** 2019-09-24

**Authors:** Rizwana Arif, Tayaba Mazhar, Mashal Jamil

**Affiliations:** 1 Obstetrics and Gynecology, Khyber Teaching Hospital, Peshawar, PAK; 2 Obstetrics and Gynecology, Rehman Medical Institute, Peshawar, PAK

**Keywords:** misoprostol, dinoprostone, labor induction, primigravida, full term pregnancy

## Abstract

Objective

The objective of this study was to compare the effectiveness of vaginally administered misoprostol to that of vaginally administered dinoprostone at six-hour intervals in a well-homogenized cohort of full-term, nulliparous women with an unfavorable cervix and without any pregnancy complications.

Materials and methods

A cohort of 100 nulliparous women at more than 40 weeks of gestation was included in this study. The primary outcomes to be measured were induction to delivery interval and incidence of vaginal births within 12 and 24 hours. Neonatal intensive care unit admissions for poor neonatal outcomes and obstetrical complications were secondary outcomes.

Results

A significant reduction in the induction to delivery interval was observed in the misoprostol group as compared to the dinoprostone group (10.2 ± 0.8 vs. 16.5 ± 0.7, p < 0.001). More women in the misoprostol group delivered within 12 hours as compared to the dinoprostone group (30 [60%] vs. 17 [34%], p < 0.001) and within 24 hours (48 [96%] vs. 39 [78%], p < 0.05). In the misoprostol group, spontaneous rupture of the membranes occurred more frequently (46 [92%] vs. 35 [70%], p < 0.05) with less need for oxytocin augmentation during labor (14% vs. 30%, p < 0.05). A significant reduction in additional dose requirement (7.5% vs. 22%, p < 0.05) and a lower rate of Caesarean section was observed in the misoprostol group (6% vs. 24%, p < 0.04). A statistically insignificant low Apgar score was noted in the dinoprostone group compared to the misoprostol group.

Conclusion

Vaginally administered misoprostol is more effective than vaginally administered dinoprostone at six-hour intervals in nulliparous women beyond 40 weeks of gestation without pregnancy complications.

## Introduction

The rate of induction has been reported as more than 20%, depending upon various centers [1,2]. Intentional initiation of labor for delivery in the fetomaternal unit before spontaneous onset is termed as labor induction [3,4]. It is carried out for fetomaternal indications, the most common being prolonged pregnancy [5]. Continuing pregnancy beyond 41 weeks can have a risk to both the fetus and the mother [6,7]. Therefore, studies have suggested induction of labor rather than continuing the pregnancy [8,9]. Cervical ripening is required to increase the chances of successful induction of labor when the Bishop score is low [3,4,10-12]. Studies have shown the effectiveness of vaginal prostaglandins for cervical ripening.

Dinoprostone, which is prostaglandin F2, is an FDA-approved drug that leads to cervical ripening, initiates uterine contractions, and thus facilitates vaginal delivery. Misoprostol, which is prostaglandin E1, is currently not FDA-approved for labor induction, but it has the advantages of low cost, higher efficacy, and no need for lower temperature storage. Vaginally administered misoprostol is more potent in comparison to dinoprostone [11].

Misoprostol has been used in various randomized studies for cervical ripening and labor induction, and these studies have concluded that misoprostol is effective but is at the expense of tachysystole and uterine hyperstimulation [4,13-17]. However, no proper evaluation of misoprostol in comparison to dinoprostone has been done in nulliparous women at term gestation with an unfavorable cervix, which is a group of women that contribute to a higher Caesarean section rate. Meconium staining of amniotic fluid, fetal heart rate (FHR) variabilities, and uterine hyperstimulation are the potential complications of vaginally administered misoprostol [16]. Studies have reported that during prolonged pregnancy nulliparity, unfavorable Bishop score, and epidural analgesia were the parameters, rather than induction of labor, that would increase the odds of having a Caesarian section rather than normal vaginal delivery [18]. Therefore, we conducted this study to compare the efficacy of vaginally administered misoprostol (50 mcg) to that of vaginally administered dinoprostone (3 mg), administered at six-hour intervals in a well-homogenized cohort of full-term nulliparous women with an unfavorable cervix and without pregnancy complications.

## Materials and methods

This study was conducted at the Gynaecology and Obstetrics Unit “C” of the Gynaecology and Obstetrics Department of Khyber Teaching Hospital, a tertiary care hospital with 4000 deliveries a year with major referrals from peripheries of the Khyber Pakhtunkhwa. One hundred women were recruited for the study from January 1, 2018, to December 31, 2018. The main indication was prolonged pregnancy after 40 weeks of gestation. The Ethical Committee of the hospital approved the study, and informed consent was obtained from the patients who were included in the study. Prostaglandin was vaginally administered by one of the trained medical officers on duty, who was responsible for managing these women during labor. Both the patient and the trained medical officer were double-blinded to the study to reduce selection bias.

Inclusion criteria were primigravida >18 years, dating scan during the first trimester for the estimated date of delivery, cephalic presentation, Bishop score of ≤ 6 suggesting unfavorable cervix, and intact membranes. Exclusion criteria were a contraindication to prostaglandins, abnormal placenta, previous Caesarian section, premature rupture of membranes, and multigravidity.

Gestational age was calculated from the last menstrual period and was confirmed on first trimester dating scan using crown-rump length. Prolonged labor was defined as >24 hours since last induction of labor. Uterine tachysystole was defined as a uterine contraction frequency of five or more every 10 minutes. Uterine hypertonus was defined as a single contraction lasting more than one minute, and the condition in which there is non-reassuring FHR with tachysystole or hypertonus was called uterine hyperstimulation syndrome. Fetal tachycardia (i.e., heart rate > 160 beats per minute for 10 minutes); bradycardia (heart rate below 110 beats per minute for 10 minutes); absent, late, or prolonged decelerations; or variable decelerations (i.e., heart rate falling to less than 70 beats per minute for 60 seconds) was defined a non-reassuring FHR.

Continuous variables were calculated as mean ± standard deviation. The categorical data were recorded as percentages. P-value of ≤ 0.05 was considered significant. Discrete data were compared by chi-square analysis, and comparison between the two drug therapies was made by using paired Student’s two-tailed t-test.

## Results

When the two groups were compared in terms of patient’s age (20 ± 3 vs. 21 ± 5), the p-value was statistically non-significant. The induction to delivery time was significantly shorter (p = 0.001) in patients in the misoprostol group. As compared to the dinoprostone group, the number of women who delivered within 12 hours was higher in the misoprostol group. In the misoprostol group, almost all patients delivered within 24 hours. Repeated doses were needed more in the dinoprostone group (p = 0.05). Spontaneous rupture of membrane occurred more frequently in the misoprostol group (p = 0.05). Augmentation was also needed in fewer patients in the misoprostol group (p = 0.05). Tachysystole (p < 0.05) and meconium staining of the amniotic fluid (p-value non-significant) occurred more often in the dinoprostone group as did abnormal heart rate tracing (p = 0.05), as shown in Table 1.

**Table 1:** Obstetrical parameters Abbreviation: SD, standard deviation.

	Misoprostol n=50	Dinoprostone n=50	P-value
Mean age (years)	20 ± 3	21 ± 5	0.8
Induction to delivery time (hours) ± SD	10.2 ± 0.8	16.5 ± 0.7	0.001
Delivery < 12 hours	30 (60%)	17 (34%)	0.001
Delivery < 24 hours	48 (96%)	39 (78%)	0.05
Prolonged labor^*^	1 (2%)	1 (2%)	0.1
Number of Doses			
One dose	21 (42%)	13 (26%)	0.05
Two doses	3 (7%)	7 (15%)	
Three doses	1 (2%)	5 (10%)	
Spontaneous rupture of membranes	20 (40%)	21 (42%)	< 0.05
Oxytocin use	7 (14%)	15 (30%)	< 0.05
Hyperstimulation	0	0	0.8
Tachysystole	0	5 (10%)	< 0.05
Meconium staining	1 (2%)	2 (4%)	0.7

In both groups, most women had a vaginal delivery, 92% with misoprostol, and 88% with dinoprostone. Caesarian section was almost the same in both groups (p = 0.7), as shown in Table 2.

**Table 2:** Indications for Caesarian section and mode of delivery

	Misoprostol n = 50	Dinoprostone n= 50	P-value
Spontaneous vaginal delivery	46 (92%)	35 (70%)	0.6
Instrumental vaginal delivery	1 (2%)	3 (6%)	0.4
Caesarean section	3 (6%)	12 (24%)	0.04
Abnormal fetal heart rate	2 (4%)	7 (14%)	0.05
Failure to progress in labor	0	1 (2%)	0.9

**Figure 1:**
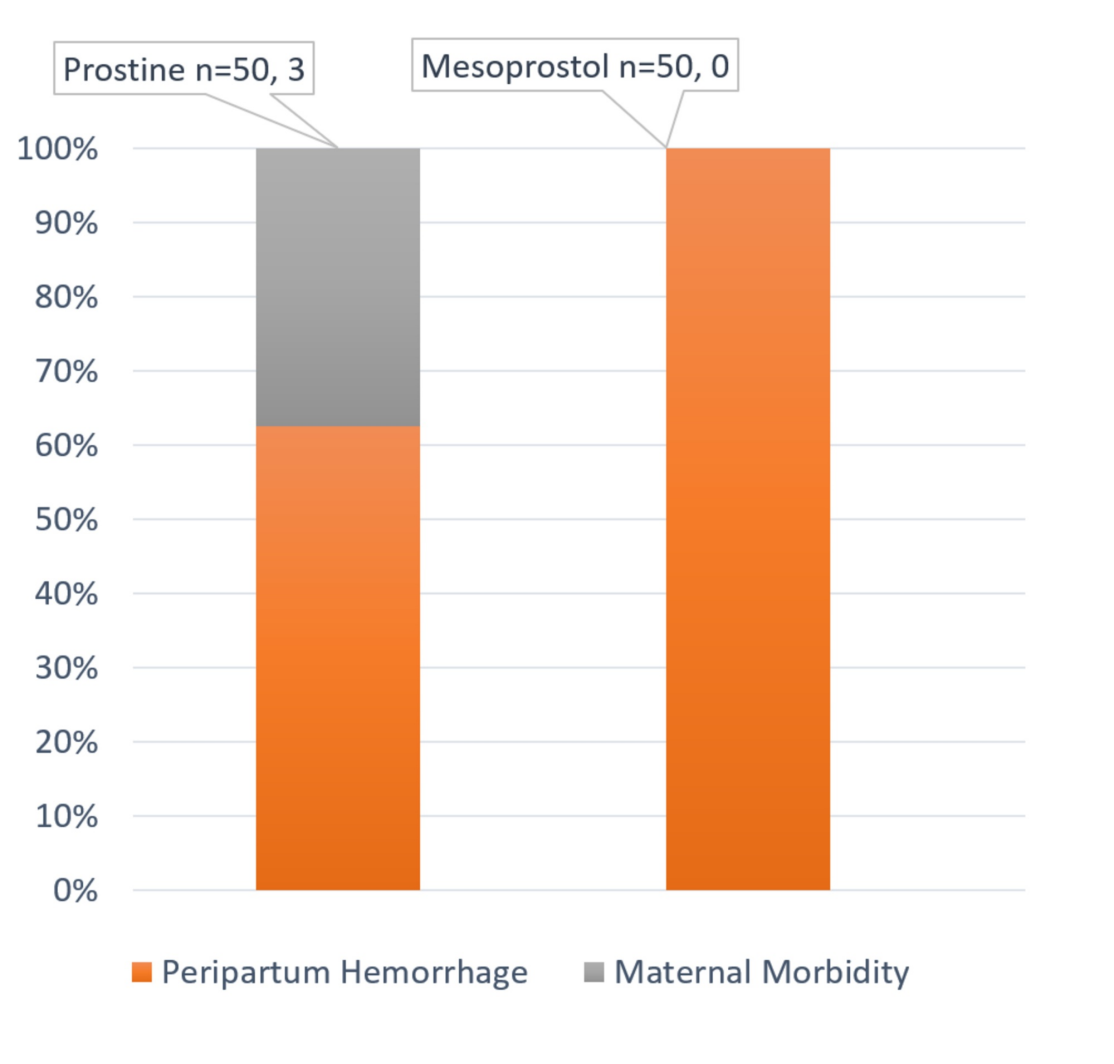
Obstetric complications

There were no uterine ruptures or maternal mortality with the use of either of the prostaglandins. Postpartum hemorrhage was higher in the misoprostol group. Morbidities related to dinoprostone included fever, as shown in Figure 1.

Birth weight was comparable in both groups. More babies in the dinoprostone group had a first-minute Apgar score less than seven and thus needed neonatal resuscitation, as shown in Table 3.

**Table 3:** Neonatal outcome

Apgar Score	Misoprostol n = 50	Dinoprostone n= 50
10	32 (64%)	18 (36%)
9	8 (16%)	14 (28%)
8	6 (12%)	10 (20%)
7	4 (8%)	8 (16%)
Mean	9.52	9.35
Standard Deviation	0.8	0.9

## Discussion

Labor induction is very much prevalent in labor suites in Pakistan and the world at large [19,20]. The indications most common are postdate pregnancy (gestation more than 41 weeks). Various agents have been used in the past, but prostaglandins E2 and F2 alpha are the most widely used drugs due to their efficacy for the termination of pregnancy. Prostaglandin PGE1 (misoprostol) has been recently introduced as a labor-inducing agent. Literature has reported its action as an inducing agent in the first trimester, but its use for the induction of labor at full term has not been studied locally or internationally. The anxiety that expectant mothers experience after their due date is another temptation for obstetricians to use prostaglandins [21]. Our study compared dinoprostone and misoprostol in primigravid patients at more than 40 weeks' gestation with intact membrane and unfavorable cervix for labor induction.

In our study, misoprostol use not only shortened the time between induction and delivery, but it also was significantly more effective than dinoprostone, needing at the most two doses. Day et al. demonstrated a significant reduction in induction to delivery time and use of oxytocin augmentation when misoprostol was compared with dinoprostone [19]. Although we have found in our study a statistically significant difference in favor of misoprostol, studies have reported concerns regarding uterine hyperstimulation and abnormal FHR tracings. Uterine hyperstimulation, tachysystole, and meconium staining have been reported in literature more with misoprostol than dinoprostone [19]. Painful labor has been associated with misoprostol use [14]. No such effects with misoprostol were recorded during our study. We had no case of fetal distress or uterine hyperstimulation needing emergency Caesarian section. Therefore, we recommend the proper selection of patients to avoid any maternal or fetal complications [21]. Similar results have been reported in a study by Akhtar et al. [22]. Papanikolaou et al. had similar results as our study, but they had more abnormal FHR tracings and more neonatal intensive care unit admissions [23]. These adverse events occur more in third-world countries where facilities for monitoring the mother and the fetus and emergency Caesarean section resources are drastically limited. Therefore, our study recommends using misoprostol, as it is cheaper and as effective as dinoprostone. Besides, when the patient is appropriately selected, the complication risks are further reduced with misoprostol than the standard dinoprostone. Therefore, misoprostol can be safely used in a third-world country like Pakistan, where the majority of the population cannot afford the cost of the management and complication of pregnancy.

## Conclusions

Compared to dinoprostone, misoprostol at a six-hour interval is highly effective for cervical ripening and for inducing labor. With proper patient selection and monitoring of patients induced with misoprostol, maternal, and fetal complications can be prevented, and optimum obstetrical outcomes can be achieved. Additionally, its low cost will make it more affordable for third-world countries such as Pakistan.
